# Negative Association between Testosterone Concentration and Inflammatory Markers in Young Men: A Nested Cross-Sectional Study

**DOI:** 10.1371/journal.pone.0061466

**Published:** 2013-04-18

**Authors:** Johannes Bobjer, Marianna Katrinaki, Christos Tsatsanis, Yvonne Lundberg Giwercman, Aleksander Giwercman

**Affiliations:** 1 Reproductive Medicine Centre, Skåne University Hospital Malmö, Malmö, Sweden; 2 Reproductive Medicine Research Group, Department of Clinical Sciences Malmö, Lund University, Malmö, Sweden; 3 Department of Clinical Chemistry, School of Medicine, University of Crete, Medical School,Heraklion, Crete, Greece; 4 Molecular Genetic Reproductive Medicine, Department of Clinical Sciences Malmö, Lund University, Malmö, Sweden; German Diabetes Center, Leibniz Center for Diabetes Research at Heinrich Heine University Duesseldorf, Germany

## Abstract

**Objective:**

Low grade systemic inflammation (LGSI) as well as androgen deficiency has in older men been associated with several pathologies, including cardiovascular disease (CVD). We wanted to investigate whether low testosterone levels are linked to biomarkers of LGSI already in young age, before any concurrent manifestations of CVD or other systemic diseases.

**Design:**

Nested cross-sectional study.

**Methods:**

Forty subfertile biochemically hypogonadal (n = 20) or eugonadal (n = 20) men (mean age 37 years, SD = 4.3) and 20 age-matched controls were randomly selected from an ongoing study on male subfertility. Subjects comprised male partners in infertile couples in whom also subnormal sperm concentration was present. Blood sampling, interviews, and anthropometric measures were undertaken. Serum levels of testosterone, LH, estradiol, SHBG, and 21 LGSI-markers were assessed.

**Results:**

Among 21 inflammatory markers, macrophage inflammatory protein 1-alpha (MIP1a) (ß = −0.025; p = 0.028), 1-beta (MIP1B) (ß = −0.015; p = 0.049) and tumor necrosis factor alpha (TNFa) (ß = −0.015; p = 0.040) showed negative association to total testosterone (TT) levels. MIP1a (ß = −1.95; p = 0.001) and TNFa (ß = −0.95; p = 0.014) showed negative association to calculated free testosterone (cFT) levels. Compared to men with normal TT and cFT levels, TNFa levels were higher in men with subnormal levels of TT (mean ratio 1.61; p = 0.006) and cFT (mean ratio 1.58; p = 0.007). Also, MIP1a levels were higher in men with subnormal levels of TT (mean ratio 1.84; p = 0.030).

**Conclusions:**

Subnormal testosterone may already in young age associate to LGSI, which might be a part of the mechanism underlying adverse health outcomes of male hypogonadism.

## Introduction

Androgen deficiency in older men has been associated with the occurrence of cardiovascular disease (CVD) [1], type 2 diabetes [2], dyslipidemia [3] and the metabolic syndrome (MetS) [4]. These conditions have also been linked to low grade systemic inflammation (LGSI), characterized by increased levels of pro-inflammatory cytokines and chemokines in serum [5]. These inflammatory mediators participate in the activation of innate and adaptive immune cells and contribute to tissue damage, insulin resistance [6] and atherosclerosis. LGSI is partly the result of sustained activation of monocytes in the periphery that differentiate to macrophages, adsorb oxidized lipoproteins [7], migrate through the endothelial barrier to the intima-media layer of arteries and form foam cells - the primary component of atherosclerotic lesions [8]. Stimulation from cytokines also leads to activation of endothelial cells and enhanced expression of adhesion molecules that facilitates attachment of macrophages.

In elderly men, low levels of endogenous androgens increase the risk of atherosclerosis [9] and is related to the progression of intima-media thickness of the common carotid artery [10]. Furthermore, testosterone levels inversely correlate to degree of coronary atherosclerosis [11]. Based on a meta-analysis including 19 studies, Ruige *et al* found weak association between endogenous testosterone and risk for CVD in elderly but not middle-aged men and raise the question whether low androgens are a cause of CVD or, rather, a marker of a poor general health [12].

Whether low androgen levels contribute to LGSI and eventually CVD or whether general metabolic dysregulation leading to both LGSI and hypogonadism, has been a matter of discussion [13]. Higher prevalence of autoimmune diseases in women and androgen deficient men suggests an immunosuppressive action of testosterone [14], as do experimental studies in which testosterone was found to attenuate inflammatory cytokines [15] and to up-regulate anti-inflammatory cytokines [16]. Moreover, significant improvement in the levels of inflammatory markers following androgen replacement therapy in hypogonadal men and hypogonadal men with MetS has been shown [17], [18].

However, previous studies linking CVD, MetS, LGSI and hypogonadism have exclusively focused on older men and a limited number of inflammatory mediators. We hypothesized that men with low testosterone, but without manifestations of systemic disease, already in young age may have higher levels of inflammatory markers. To investigate this, we analyzed an array of cytokines and chemokines in sera from 60 young men, who were either hypogonadal or eugonadal.

## Subjects and Methods

### Ethics Statement

The study was approved by the regional ethical review board at Lund University, Sweden. All subjects provided written informed consent.

### Study Design

Subjects for this nested study were selected among participants of an ongoing case-control study on hypogonadism among men seeking medical care due to subfertility. A free online random number generator (random.org) was used to identify 20 hypogonadal subfertile men, 20 eugonadal subfertile men and 20 controls. Hypogonadism was strictly biochemically defined according to our laboratory’s normal levels as testosterone <8.0 nmol/L and/or LH >8.6 IU/L [19]. No men, in any of the groups, received androgen replacement therapy. Mean levels of the hormones analyzed are presented in table 1.

**Table 1:** Median levels of inflammatory markers in the two subgroups of subfertile men (hypo- and eugonadal) as compared to the 20 controls.

	All (n = 60)			Subfertile		Controls (n = 20)						
				HG (n = 20)	EG (n = 20)							
Cytokine/Chemokine	Median	Min	Max	Median	Median	Median	*P* crude, HG vs. contr.	*P* adj, HG vs. contr.	*P* crude, EG vs. contr.	*P* adj, EG vs. contr.	*P* crude, HG vs. EG	*P* adj, HG vs. EG
IL8 (pg/mL)	9.20 (5.61–17.3)	1.20	203	9.31 (5.88–15.6)	8.71 (5.61–17.3)	9.31 (5.37–26.0)	NS	NS	NS	NS	NS	NS
IL12p70 (pg/mL)	3.44 (0.40–21.0)	0.40	1099	3.78 (0.40–17.4)	2.77 (0.77–19.0)	4.46 (0.40–27.0)	NS	NS	NS	NS	NS	NS
IL17 (pg/mL)	1.90 (0.34–6.47)	0.2	182	1.51 (0.25–3.75)	2.33 (0.40–7.76)	2.39 (0.39–7.73)	NS	NS	NS	NS	NS	NS
EGF (pg/mL)^a^	140 (94.7–254)	39.2	640	130 (79.3–234)	194 (117–287)	130 (94.3–199)	NS	NS	NS	*	NS	NS
FGF2 (pg/mL)	22.0 (3.47–45.2)	1.8	259	17.0 (1.80–40.8)	28.9 (8.46–47.7)	22.6 (8.46–54.0)	NS	NS	NS	NS	NS	NS
IFNG (pg/mL)	2.73 (0.75–13.7)	0.1	288	3.09 (0.31–10.1)	2.00 (0.75–20.4)	3.33 (1.07–23.3)	NS	NS	NS	NS	NS	NS
IP10 (pg/mL)	199 (152–249)	52.9	1021	168 (145–328)	182 (154–232)	213 (171–254)	NS	NS	NS	NS	NS	NS
MCP-1 (pg/mL)	488 (383–587)	199	1269	496 (402–666)	472 (384–570)	479 (340–610)	NS	NS	NS	NS	NS	NS
MIP1a (pg/mL)	15.2 (8.61–26.6)	3.50	102	17.6 (5.40–26.6)	14.3 (10.0–20.4)	17.2 (9.04–35.5)	NS	NS	NS	NS	NS	NS
MIP1B (pg/mL)	50.8 (36.4–71.7)	12.0	296	52.0 (37.0–70.8)	52.1 (36.8–81.3)	45.3 (27.3–69.0)	NS	NS	NS	NS	NS	NS
TNFa (pg/mL)	7.93 (5.85–11.7)	2.23	42.1	8.74 (6.14–18.3)	8.35 (4.84–11.6)	7.83 (5.85–9.43)	NS	NS	NS	NS	NS	NS
hs-CRP (mg/L)	0.10 (0.04–0.17)	0.02	2.23	0.15 (0.06–0.47)	0.11 (0.05–0.15)	0.05 (0.02–0.11)	**	**	*	*	NS	NS

Data are median (interquartile range) due to non-Gaussian distribution. Univariate regression analysis after log-transformation. *P* adj refers to adjustment for age and BMI. Non-detectable levels of inflammatory markers and levels below the lower detection limit of the assay (Table S1) were replaced by a value corresponding to the lower detection limit (assay sensitivity). HG, hypogonadal; EG, eugonadal; IL1B, interleukin 1-beta; IL1ra, interleukin 1-receptor antagonist; IL, interleukin; EGF, epithelial growth factor; FGF2, fibroblast growth factor 2; IFNG, interferon gamma; IP10, interferon gamma-induced protein 10; MCP1, monocyte chemotactic protein 1; MIP1a, macrophage inflammatory protein 1-alpha; MIP1B, macrophage inflammatory protein 1-beta; TNFa, tumor necrosis factor alpha; hs-CRP, high sensitive c-reactive protein;

^a^, one missing sample,

* , *P*<0.05;

** , *P*<0.01.

The primary subfertility cohort comprised a consecutive group of men from couples unable to conceive after one year of regular sexual activity, in the absence of an explanatory female factor (ovulatory disturbance, tubal obstruction and/or endometriosis).

Furthermore, following inclusion criteria were applied: age 18–50 years at inclusion and sperm concentration <20 million/mL. Of the 286 eligible men, 115 (40%) did not reply despite a reminder by letter, 52 (18%) declined to take part in the study, and 119 (42%) gave their consent.

The control group was selected from the Swedish Population Register by matching their date of birth with that of the subfertile men. One hundred men were included at the time of selection of subjects for the present study. None of the selected controls were receiving any fertility treatment.

### Inclusion

Physical examination including assessment of height (without shoes, with a stadiometer to the nearest 1 mm) and weight (in light clothing, with an electronic scale to the nearest 0.1 kg) was undertaken. Information on other diseases as well as active medication was retrieved from interviews at inclusion. None of the participants reported any serious medical illness, apart from the subfertility problem. One subjected had Klinefelter syndrome, one reported two previous occasions of epididymitis, one had had mumps orchitis as a child and yet one was unilateral orchidectomized due to tuberculosis in childhood. One subject was on current lipid lowering therapy and two were on antihypertensive treatment. Neither therapy was discontinued prior to inclusion. None of the subjects or controls was on any anti-diabetic/insulin therapy, opioids, glucocorticoids or other anti-inflammatory drugs.

### Hormone Assessment

Fasting blood samples were drawn between 8 and 10 am from all participants. Hormone levels in plasma were assessed at the Dept. of Clinical Chemistry, Skåne University Hospital, Malmö, Sweden.

Serum values of total testosterone (TT) were assessed by a two-step competitive immunoassay with a luminometric technique (Electro Chemi Luminiscence Immunoassay (ECLI); lower detection level 0.087 nmol/L; imprecision (CV%), 2.4% at 1.9 nmol/L and 4.0% at 25.5 nmol/L). Luteinizing hormone (LH) and sex hormone binding globulin (SHBG) concentrations were determined with a one-step immunometric sandwich assay with a luminometric technique (ECLI); (P-LH) lower detection level 0.10 IU/L; CV%, 2.0% at 5.0 IU/L and 2.2% at 55.2 IU/L; (SHBG) LDL 0.35 nmol/L; CV%, 1.0% at 22.0 nmol/L and 1.1% at 49.5 nmol/L. Estradiol concentrations in serum were assessed by an immunofluorometric method (DELFIA Estradiol, Wallace OY; lower detection level 8 pmol/L; CV%, 20% at 30 pmol/L and 10% at 280 pmol/L. Free testosterone was calculated from total testosterone, SHBG and fixed albumin levels [20].

### Inflammatory Markers

Inflammatory markers in serum were analyzed at the Dept. of Molecular Biology, School of Medicine, University of Crete, Heraklion, Crete, Greece. Sera were tested for the levels of tumor necrosis factor alpha (TNFa), macrophage inflammatory protein 1 alpha (MIP1a), 1 beta (MIP1B), monocyte chemotactic protein 1 (MCP1), endothelial growth factor (EGF), fibroblast growth factor 2 (FGF2), interferon gamma (IFNG), interleukin 12p40 (IL12p40), IL12p70, IL1B, interleukin 1 receptor antagonist (IL1RA), IL4, IL6, IL7, IL10, IL13, IL17, interferon gamma-induced protein 10 (IP10), IL9 and IL8, using a bead-based immunoassay (Lincoplex-Millipore) and analyzed using Luminex apparatus (Luminex, USA) as recommended by the manufacturer and previously described [21]. Assay sensitivity, accuracy, inter- and intra-assay precision for all inflammatory markers are presented in Table S1 (also available at http://www.millipore.com/userguides/tech1/proto_hcyto-60k). C-reactive protein (CRP) (high sensitivity) was analyzed in a Beckman AU5400 analyzer using Beckman reagents (lower detection level 0.02 mg/L; inter assay precision (CV%), 7.5%; intra assay precision (CV%), 5.0%).

### Statistical Analysis

Non-detectable levels of inflammatory markers and levels below the lower detection limit of the assay were replaced by a value corresponding to the lower detection limit (Table S1). First, we made descriptive statistics of all inflammatory markers (Table 1). Then, we log-transformed the inflammatory marker levels in order to obtain normal distributions of the residuals. Univariate regression models were used for testing associations between study variables and differences between groups. In these analyses we excluded inflammatory markers (IL1B, IL1RA, IL4, IL6, IL7, IL9, IL10, IL12p40 and IL13) which had ≥33% non-detectable values (Table S1). Age and fertility status (categorized as subfertile vs. control) were included as covariables. Since body mass index (BMI) is negatively associated with testosterone levels, in order to not over-adjusting for the effect of low testosterone, primarily we did not include BMI in the main analysis. However, all statistically significant findings were tested for robustness to adjustment for BMI and even these data are presented.

Association between testosterone levels and inflammatory markers were also tested after excluding the three subjects on lipid lowering therapy or antihypertensive treatment.

Primarily, the association between the levels of TT, cFT and estradiol - as continuous variables - and the concentrations of inflammatory markers was explored. Thereafter, following categorizing the men in groups as having subnormal or normal testosterone using cut off levels of 8.0 nmol/L for TT and 220 pmol/L for cFT, additional analysis was made for those inflammatory markers for which the association was statistically significant.

The mean value results are presented as back transformed (antilogarithmic) geometric means. Accordingly, obtained differences between the groups correspond to mean ratios that reflect the relative differences between groups in levels of inflammatory markers.

## Results

Group characteristics including hormone levels and between groups comparisons are presented in table 2. Descriptive statistics and between groups comparisons for inflammatory markers are presented in table 1.

**Table 2:** Group characteristics and hormone levels in serum in the two subgroups of subfertile men (hypo- and eugonadal) as compared to the 20 controls.

	Subfertile		Controls (n = 20)	Total (n = 60)						
	HG (n = 20)	EG (n = 20)			*P* crude, HG vs. contr.	*P* adj, HG vs. contr.	*P* crude, EG vs. contr.	*P* adj, EG vs. contr.	*P* crude, HG vs. EG	*P* adj, HG vs. EG
Age (yr)	37.1 (3.75)	36.0 (4.88)	39.3 (8.33)	37.4 (6.04)	NS	NA	NS	NA	NS	NA
BMI (kg/m^2^)	28.0 (7.14)	26.3 (3.52)	25.1 (2.98)	26.5 (4.98)	NS	NA	NS	NA	NS	NA
TT(nmol/L)	10.3 (4.24)	13.6 (4.27)	14.3 (3.35)	12.7 (4.28)	**	*	NS	NS	*	*
SHBG(nmol/L)	31.2 (15.6)	35.1 (16.9)	33.7 (12.6)	33.3 (15.0)	NS	NS	NS	NS	NS	NS
cFT(pmol/L)	225 (95.2)	279 (67.1)	304 (63.2)	270 (82.1)	**	**	NS	NS	*	*
LH (IU/L)	10.7 (6.83)	4.82 (1.42)	3.33 (1.67)	6.29 (5.19)	**	**	NS	NS	**	**
Estradiol (pmol/L)	97.9 (28.6)	101 (24.6)	97.0 (28.9)	98.8 (27.0)	NS	NS	NS	NS	NS	NS

All data are presented as means (SD). Univariate regression analysis. *P* adjusted refers to the factors age, BMI and fertility status. HG, hypogonadal; EG, eugonadal; BMI, body mass index; TT, total testosterone; SHBG, sex hormone binding globuline; cFT, calculated free testosterone; LH, luteinizing hormone; NS, not significant; NA, not applicable;

* , *P*<0.05;

** , *P*<0.01.

### Association of TT, cFT and Estradiol with Inflammatory Markers in Serum

**Figure 1:**
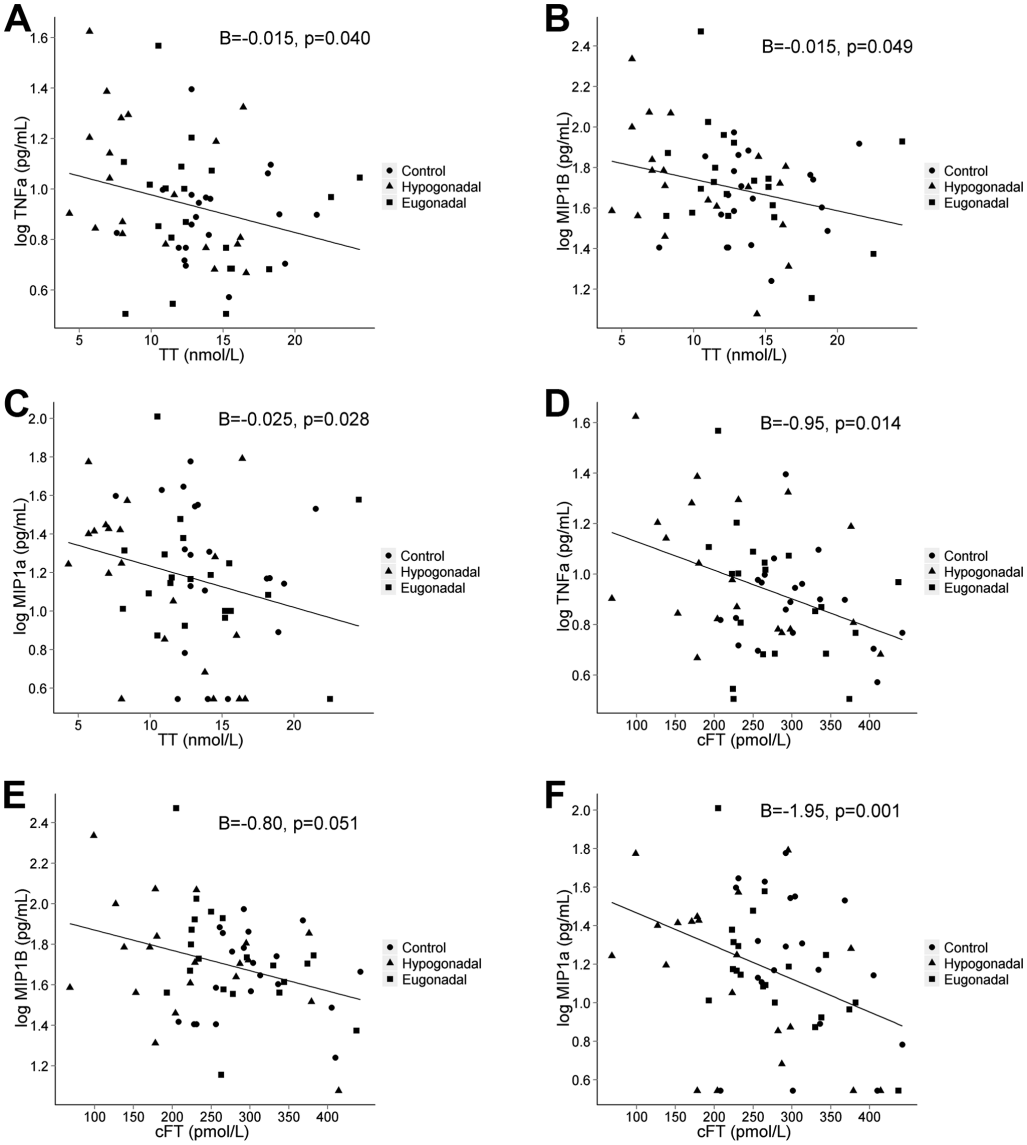
Associations between TT and (A) TNFa, (B) MIP1B, (C) MIP1aand between cFT and (D) TNFa, (E) MIP1B, (F) MIP1a. All inflammatory markers are log values. B represents regression coefficients derived from univariate regression analysis adjusted for age and fertility status. Initial grouping of men indicated in figure as subfertile hypogonadal (n = 20), subfertile eugonadal (n = 20) and controls (n = 20). TT, total testosterone; cFT, calculated free testosterone; TNFa, tumor necrosis factor alpha; MIP1B, macrophage inflammatory protein 1−beta; MIP1a, macrophage inflammatory protein 1-alpha.

MIP1a (ß = −0.025; 95% CI, −0.048, −0.003; p = 0.028), MIP1B (ß = −0.015; 95% CI, −0.030, −0.001; p = 0.049) and TNFa (ß = −0.015; 95% CI, −0.029, −0.001; p = 0.040) showed negative association to TT levels. MIP1a (ß = −1.95; 95% CI, −3.09, −0.80; p = 0.001) and TNFa (ß = −0.95; 95% CI, −1.69, −0.20; p = 0.014) showed negative association to cFT levels (Fig. 1). After adjustment for BMI, the negative association of cFT to TNFa (p = 0.019) and MIP1a (p = 0.002) remained statistically significant. Also, EGF showed positive association to TT levels (p = 0.046), only when adjustment included BMI. No other statistically significant associations between TT, cFT, estradiol and inflammatory markers were found. All associations are presented in table 3. Risk estimates remained unchanged after exclusion of the three subjects on lipid lowering therapy or antihypertensive treatment (data not shown).

**Table 3:** Associations between levels of inflammatory markers and total and free testosterone as well as estradiol concentrations in serum.

		TT				cFT				E2			
Cytokine/Chemokine	n	B	95% CI	*P* adj	*P* adj BMI	B	95% CI	*P* adj	*P* adj BMI	B	95% CI	*P* adj	*P* adj BMI
IL8	60	−0.019	−0.043, 0.004	0.105	0.363	−1.17	−2.43, 0.096	0.069	0.095	−0.002	−0.005, 0.002	0.403	0.233
IL12p70	60	−0.047	−0.11, 0.012	0.115	0.119	−1.97	−5.13, 1.20	0.218	0.232	0.001	−0.008, 0.010	0.825	0.864
IL17	60	−0.014	−0.060, 0.031	0.533	0.802	−0.85	−3.30, 1.61	0.493	0.554	−0.001	−0.008, 0.006	0.859	0.740
EGF	59	0.010	−0.008, 0.027	0.262	0.046*	0.25	−0.69, 1.18	0.598	0.523	0.001	−0.002, 0.003	0.613	0.830
FGF2	60	−0.016	−0.054, 0.022	0.405	0.645	−1.39	−3.40, 0.62	0.171	0.203	−0.001	−0.007, 0.005	0.731	0.612
IFNG	60	−0.028	−0.083, 0.027	0.315	0.343	−1.98	−4.91, 0.95	0.181	0.193	−0.003	−0.012, 0.006	0.479	0.447
IP10	60	−0.009	−0.022, 0.004	0.152	0.469	−0.33	−1.03, 0.36	0.344	0.435	−0.001	−0.003, 0.001	0.247	0.129
MCP1	60	0.002	−0.008, 0.012	0.674	0.508	0.18	−0.34, 0.69	0.496	0.468	0.0001	−0.001, 0.002	0.881	0.939
MIP1a	60	−0.025	−0.048, −0.003	0.028*	0.068	−1.95	−3.09, −0.80	0.001**	0.002**	−0.003	−0.007, 0.001	0.097	0.058
MIP1B	60	−0.015	−0.030, −0.001	0.049*	0.220	−0.80	−1.60, 0.002	0.051	0.071	0.0001	−0.003, 0.002	0.763	0.509
TNFa	60	−0.015	−0.029, −0.001	0.040*	0.212	−0.95	−1.69, −0.20	0.014*	0.019*	0.0001	−0.003, 0.002	0.685	0.420
hs-CRP	60	−0.021	−0.051, 0.008	0.148	0.658	−0.95	−2.53, 0.63	0.233	0.321	0.001	−0.003, 0.006	0.599	0.896

All inflammatory markers have been log-transformed prior to analysis. All calculations are adjusted for age and fertility status, and when indicated also for BMI. Univariate regression analysis. CI, confidence interval; BMI, body mass index; TT, total testosterone; cFT, calculated free testosterone; E2, estradiol; IL, interleukin; EGF, epidermal growth factor; FGF2, fibroblast growth factor; IFNG, interferon gamma; IP10, interferon gamma-induced protein 10; MCP1, monocyte chemotactic protein 1; MIP1a, macrophage inflammatory protein 1-alpha; MIP1B, macrophage inflammatory protein 1-beta; TNFa, tumor necrosis factor alpha; hs-CRP, high sensitive c-reactive protein;

* , *P*<0.05;

** , *P*<0.01.

### Inflammatory Markers in Relation to Subnormal TT or cFT in Serum

Among the 60 included men, 12 (20%; 95% CI, 0.11, 0.32) had TT levels <8.0 nmol/L. Thirteen (22%; 95% CI, 0.12, 0.34) had cFT levels <220 pmol/L. TNFa levels were significantly higher in men with subnormal TT levels (mean ratio 1.61; 95% CI, 1.16, 2.26; p = 0.006) or cFT levels (mean ratio 1.58; 95% CI, 1.14, 2.20; p = 0.007). Also, MIP1a levels were significantly higher in men with subnormal TT levels (mean ratio 1.84; 95% CI, 1.06, 3.19; p = 0.030) (Table 4). After adjustment for BMI, TNFa levels remained significantly higher in men with subnormal TT levels (p = 0.025) and in those with low cFT levels (p = 0.02).

**Table 4:** Comparison of levels of selected inflammatory markers in men with normal and subnormal concentration of total or free testosterone in serum.

Cytokine/Chemokine	All (n = 60)	cFT <220 pmol/L (n = 13)	cFT ≥220 pmol/L (n = 47)	Mean ratio (95% CI)	*P* adj	*P* adj BMI	TT <8 nmol/L (n = 12)	TT ≥8 nmol/L (n = 48)	Mean ratio (95% CI)	*P* adj	*P* adj BMI
TNFa (pg/mL)	8.6 (1.8)	12 (1.2)	7.7 (1.1)	1.58 (1.14,2.20)	0.007**	0.02*	12 (1.2)	7.7 (1.1)	1.61 (1.16,2.26)	0.006**	0.025*
MIP1a (pg/mL)	15 (2.3)	17 (2.9)	14 (2.2)	1.21 (0.69,2.1)	0.490	0.659	23 (2.0)	14 (2.3)	1.84 (1.06,3.19)	0.030*	0.059
MIP1B (pg/mL)	50 (1.8)	55 (1.2)	47 (1.1)	1.18 (0.82,1.71)	0.371	0.634	60 (5.6)	46 (2.3)	1.30 (0.90,1.88)	0.165	0.398

All data represent back transformed logarithmic means (SD). Univariate regression analysis, adjusted for age and fertility status, and when indicated also for BMI. BMI, body mass index; TT, total testosterone; cFT, calculated free testosterone; MIP1a, macrophage inflammatory protein 1-alpha; MIP1B, macrophage inflammatory protein 1-beta; TNFa, tumor necrosis factor alpha; CI, confidence interval; *, *P*<0.05; **, *P*<0.01.

## Discussion

Our main finding was that in relatively young men, low serum testosterone concentrations were significantly associated with elevated levels of the pro-inflammatory cytokine TNFa as well as the pro-inflammatory chemokines MIP1a and MIP1B despite any other signs of LGSI or systemic disease. In addition, we observed that men with subnormal levels of testosterone had significantly higher levels of TNFa and MIP1B in serum, whereas no association between estradiol and inflammatory markers was seen.

There is only limited amount of data on possible association between hypogonadism in young males and inflammatory response. In agreement with our finding is a previous report on 14 men of corresponding age with hypogonadotropic hypogonadism, in who increased levels of TNFa and IL6 was observed upon withdrawal of androgen replacement therapy [22]. Furthermore, in a study on more than 1.500 men a dose-dependent inverse correlation of both TT and cFT with CRP levels was found after adjustment for age, BMI and other co-morbidities [23]. However, almost two-thirds of the included subjects were above the age of 40 years and almost half of them were using anti-inflammatory drugs. Other studies have suggested an immuno modulatory action of testosterone [24], [25]. However, the latter reports were based on elderly men in whom the association between low testosterone and increased levels of inflammatory markers might be confounded by already manifest CVD and hence the direction of causality difficult to disentangle. A recent publication showed that transient elevation of estradiol levels with coincident lowering of testosterone affect several pro-inflammatory markers including MIP1a in young men [26]. The results are in agreement with our present findings regarding testosterone, but in contrast we did not find any link between estradiol and markers of LGSI. Another piece of evidence indicating relation between sex hormone levels and inflammation is our recent finding of low testosterone levels predicting increased risk of rheumatoid factor negative rheumatoid arthritis in men [27].

By focusing on a cohort of relatively young men, with no manifest systemic disease apart from the subfertility, the risk of CVD being the cause of low testosterone and LGSI was significantly reduced. Thus, even though this type of study will not provide evidence of a causal relationship between low testosterone levels and LGSI, the results strongly suggests such a mechanism of action.

The initial selection used a previously described biochemical definition of hypogonadism based on low testosterone concentrations as well as high LH levels, thereby including men with compensated testicular endocrine dysfunction. Although we detected higher levels of hs-CRP in hypogonadal men as compared to controls, this increase was rather linked to subfertility *per se*, as it also was seen among subfertile eugonadal men. However, after restricting the hypogonadal group to including men with low testosterone levels only, for two of the three LGSI markers showing statistically significant association with testosterone as continuous variable, even an inter-group difference was found. These findings were robust to adjustment for fertility status and, therefore, seem related to low testosterone levels rather than to the subfertility.

Since, apart from hs-CRP, no clinically relevant reference levels for the cytokines and chemokines included in this study have been established, the proportions of subjects presenting with pathological values cannot be ascertained. However, since none of the included men had hs-CRP levels above the normal value of 3 mg/L, it seems unlikely that other concomitant inflammatory conditions were present and thereby affecting the measurements of cytokines and chemokines included in current study.

Based on the available information on the health status and medications, we do not assume any selection bias in selection of the study group. However, the limitation of this study is the small sample size. Thus, given the present observation of a trend in several markers with non-significant associations to testosterone, including more subjects could confirm our results and possibly also show association between testosterone and other LGSI markers. Another limitation to the study is the bead-based assay that was used to analyze LGSI markers, which was not validated against ELISA. Also, a large number of individuals had low or undetectable levels of several LGSI-markers, which may account to the low sensitivity of the assay for these markers or to the fact that the population studied was apparently relatively healthy. Subsequently these markers were not included in further statistical analysis.

Visceral adiposity is well established as a source of LGSI. Thus, it needs to be considered whether high BMI might be the link between low testosterone levels and LGSI. In the main analysis we did not adjust for the effect of BMI since this parameter is closely associated with testosterone levels and inclusion of BMI might imply over-adjustment and thereby false negative results. However, four of the eight statistically significant associations were robust to inclusion of BMI as covariate. This was particularly valid for those related to cFT, which is a parameter that is corrected for BMI dependent SHBG levels [28]. Thus, our results indicate that the connection between low testosterone and inflammatory markers is not solely dependent on the amount of adipose tissue, but may also be an independent effect of androgens.

Performing multiple testing, as done in this study, implies some risk of by chance statistically significant findings. However, the fact that the association between hypogonadism and LGSI was previously reported in older subjects, stresses the biological significance of our observations.

Although our study was not designed to elucidate the mechanism behind increased risk of CVD as one of long term complications from male hypogonadism, the well recognized role of inflammation in atherosclerosis and plaque vulnerability [29] point to the associations enlightened by this study as a possible link.

In conclusion, we found higher levels of important pro-inflammatory biomarkers in young men with subnormal testosterone concentrations in the absence of concurrent metabolic disease. These findings indicate that testosterone levels are directly linked to LGSI, which in the long run may contribute to development of several adverse health effects previously associated to androgen deficiency. Moreover, in a panel of potential LGSI markers, our study has highlighted TNFa, MIP1a and MIP1B as those most strongly associated with low serum testosterone in men not suffering of serious systemic disease.

## Supporting Information

Table S1 Assay precision, accuracy, sensitivity and numbers of samples with non-detectable values for inflammatory markers. IL1B, interleukin 1-beta; IL1ra, interleukin 1-receptor antagonist; IL, interleukin; EGF, epithelial growth factor; FGF2, fibroblast growth factor 2; IFNG, interferon gamma; IP10, interferon gamma-induced protein 10; MCP1, monocyte chemotactic protein 1; MIP1a, macrophage inflammatory protein 1-alpha; MIP1B, macrophage inflammatory protein 1-beta; TNFa, tumor necrosis factor alpha, ^a^, one missing sample, ^b^, inflammatory markers with ≥33% of the samples having non-detectable values.
(DOC)
