# Supplementary material for: Negative Association between Testosterone Concentration and Inflammatory Markers in Young Men: A Nested Cross-Sectional Study
Source: PLoS One. 2013 Apr 18;8(4):e61466. doi: 10.1371/journal.pone.0061466 (PMC3630214; doi:10.1371/journal.pone.0061466)
Supplement: Table S1 — Assay precision, accuracy, sensitivity and numbers of samples with non-detectable values for inflammatory markers. IL1B, interleukin 1-beta; IL1ra, interleukin 1-receptor antagonist; IL, interleukin; EGF, epithelial growth factor; FGF2, fibroblast growth factor 2; IFNG, interferon gamma; IP10, interferon gamma-induced protein 10; MCP1, monocyte chemotactic protein 1; MIP1a, macrophage inflammatory protein 1-alpha; MIP1B, macrophage inflammatory protein 1-beta; TNFa, tumor necrosis factor alpha, a, one missing sample, b, inflammatory markers with ≥33% of the samples having non-detectable values. (DOC) [file pone.0061466.s001.doc]

| Cytokine/ Chemokine | Inter-assay precision (%CV) | Intra-assay precision (%CV) | Accuracy | Assay sensitivity (pg/mL) | Number of samples with non-detectable values (n) |
| --- | --- | --- | --- | --- | --- |
| IL1B | 7.0 | 6.1 | 100.1 | 0.4 | 36^b^ |
| IL1RA | 6.0 | 4.6 | 101.5 | 2.9 | 32^b^ |
| IL4 | 3.7 | 5.0 | 99.8 | 0.6 | 33^b^ |
| IL6 | 11.6 | 8.1 | 100.0 | 0.3 | 20^b^ |
| IL7 | 8.3 | 4.9 | 99.8 | 1.8 | 29^b^ |
| IL8 | 11.6 | 7.1 | 101.9 | 0.2 | 0 |
| IL9 | 8.4 | 2.4 | 100.8 | 0.7 | 40^b^ |
| IL10 | 13.2 | 11.0 | 99.7 | 0.3 | 30^b^ |
| IL12p40 | 12.7 | 7.2 | 124.2 | 10.5 | 42^b^ |
| IL12p70 | 14.3 | 8.7 | 100.4 | 0.4 | 17 |
| IL13 | 10.4 | 9.4 | 100.2 | 0.4 | 29^b^ |
| IL17 | 8.9 | 4.8 | 97.2 | 0.2 | 10 |
| EGF^a^ | 15.2 | 7.8 | 111.2 | 2.7 | 0 |
| FGF-2 | 12.8 | 7.5 | 100.7 | 1.8 | 15 |
| IFNG | 5.8 | 4.6 | 99.2 | 0.1 | 8 |
| IP10 | 11.2 | 4.7 | 99.0 | 1.2 | 0 |
| MCP1 | 12.0 | 6.1 | 100.7 | 0.9 | 0 |
| MIP1a | 14.4 | 5.7 | 102.8 | 3.5 | 8 |
| MIP1B | 10.6 | 5.3 | 99.3 | 4.5 | 0 |
| TNFa | 15.9 | 10.5 | 98.7 | 0.1 | 0 |
